# Diverse Recruitment for Clinical Trials Using Three Diversity-Enhancing Interventions: Qualitative Study

**DOI:** 10.2196/86541

**Published:** 2026-08-07

**Authors:** Erin Rose Cruz, Tenzin Yeshi Wangdak Yuthok, Anekwe Onwuanyi, Nicholas Vesom, Kira Clark, Phenesse Dunlap, Deidre Amaka Okeke, Farmaan Judge, Victor Ritter, FeiFei Qin, Sa Shen, Anny Rodriguez, Eldrin Lewis, Latha Palaniappan, Cati Brown-Johnson

**Affiliations:** 1 School of Medicine, Stanford Medicine, 453 Quarry Rd, Palo Alto, CA, 94304, United States, 1 650-723-2300; 2 Morehouse School of Medicine, Atlanta, GA, United States; 3 Clinical Trials Postdoctoral Research Fellowship, Strategically Focused Research Network on the Science of Diversity, American Heart Association, Dallas, TX, United States

**Keywords:** cardiovascular diseases, community networks, digital health, diversity, equity, inclusion, internet, registries, research design, social media

## Abstract

**Background:**

Lack of diversity in clinical trials reduces the generalizability of treatment efficacy across different demographic groups, thereby diminishing the relevance, quality, and equity of research findings.

**Objective:**

This study aimed to evaluate the implementation of 4 recruitment strategies and their impact on clinical trial enrollment through the Trial of Sites to Increase Diversity in Clinical Trials (TOTAL) project, a collaboration between Stanford and Morehouse School of Medicine funded by the American Heart Association.

**Methods:**

Clinical trials were randomized to 1 of 3 recruitment strategy intervention arms (community ambassador, social media ads, or registry and patient engagement platforms) or the control arm (usual recruitment). The Stanford Lightning Report, a rapid qualitative approach, was used to gather insights through interviews with principal investigators (PIs) and trial staff at baseline, 3 months, and trial completion.

**Results:**

Trial teams were supportive of increasing diversity and found the recruitment platforms generally user-friendly. External recruitment support was valued, and some trials reported improved access to participants outside their usual pool. Community ambassadors helped connect trials with community and physician networks and, in some cases, increased recruitment of Asian American participants. Registry and patient engagement platforms were easy to use and supported outreach, including direct emails to potential participants. Social media ads, particularly after the transition to BuildClinical (BuildClinical LLC), improved screening efficiency by prescreening candidates and producing a manageable number of eligible leads. Usual recruitment relied mainly on physician referrals and benefited from trusted clinician-patient relationships, but many trials exhausted their typical participant pools. Across strategies, major barriers included limited time, staff capacity, and financial resources; scarcity of diverse local populations; restrictive eligibility criteria; lack of language access; transportation burdens; and limitations of remote engagement. Additional operational barriers included institutional review board (IRB) delays, contract and vendor approval processes, and platform policy restrictions for digital advertising. Feedback from Lightning Reports led to several real-time changes, including a shift from in-house social media outreach to specialized digital recruitment support.

**Conclusions:**

Increasing diversity in clinical trials requires sustained financial, human, and institutional support, as well as effective recruitment strategies. Rapid qualitative feedback can identify implementation problems early and support real-time improvements.

## Introduction

Lack of diversity in clinical trials impacts the generalizability of treatment efficacy across demographic groups [1], undermining health equity and perpetuating disparities by restricting research findings [2]. Improving representation is essential for producing evidence that better reflects the populations most affected by disease and for reducing inequities in research participation and outcomes. More inclusive trials strengthen the scientific validity and real-world relevance of treatment findings [3]. In addition, social media recruitment can help reach underrepresented populations, but it should be used carefully because ethical concerns such as privacy, consent, and fairness depend on the study context [4]. To address this gap, Stanford and Morehouse School of Medicine collaborated on The Trial of Sites to Increase Diversity in Clinical Trials (TOTAL) project by exploring the impact of recruitment strategies on increased diverse participant enrollment.

Various strategies have been used to increase diversity in clinical trials. The TOTAL project, funded through the American Heart Association, focused on 3 strategies: community ambassadors (CAs), registries, and social media advertising. CAs can play a critical role in recruitment for clinical trials by serving as trusted representatives (eg, family or community members) who can effectively engage underrepresented groups. Their involvement can help bridge the gap between clinical research and the communities it aims to serve, thereby enhancing participation rates among minorities and vulnerable populations [5]. Registries serve as comprehensive databases that can facilitate the identification and engagement of potential participants from various demographic backgrounds, thereby addressing the long-standing issue of underrepresentation in clinical research [6,7]. Finally, social media platforms can significantly improve awareness and participation in clinical trials, particularly among minority groups who may not be adequately represented through traditional recruitment methods [8]. Social media, however, may also pose challenges, including potential biases in the populations reached and concerns regarding privacy and data security [9,10].

Granted by the American Heart Association, Stanford and Morehouse School of Medicine collaborated as part of the DIVERSE Network’s TOTAL project to expand knowledge around diverse recruitment by comparing various recruitment strategies across trials nationally. Though well known that each of these strategies (CAs, registries, and social media ads) can be beneficial, collectively, we know less about specific best practices for how these strategies get operationalized. This is what implementation science refers to as the “implementation gap,” the gap between what needs to be done (use diversity-increasing strategies for clinical trials) and what actually happens [11,12]. Evidence-based strategies can often fall short due to various barriers, including organizational culture, resource limitations, and lack of training among practitioners [13]. This study focuses specifically on understanding best practices for implementing diversity-enhancing strategies, thereby reducing the implementation gap to increase diversity in clinical trials.

## Methods

### Overview

This quality improvement study used a qualitative design to explore clinical trial stakeholders’ perspectives on diversity-enhancing recruitment strategies within the context of the TOTAL project, a larger cluster randomized controlled trial evaluating several recruitment interventions in adult cardiometabolic clinical trials across the United States. We conducted semistructured interviews with principal investigators (PIs) and clinical trial staff involved in participating studies, focusing on trial resources, accessibility, staff capacity, expertise, and responsiveness to local populations. Interview data were analyzed using a rapid qualitative approach, the Stanford Lightning Report framework, in which multiple coders applied consensus coding to identify key themes. Ethical approval was obtained from Stanford’s Institutional Review Board (IRB), with an exemption granted for the qualitative interview component. The detailed project (including recruitment and intervention, and data collection and analysis) is described below.

### Setting: TOTAL Project

#### TOTAL Project Recruitment

The TOTAL project was conducted as a cluster randomized controlled trial using a mixed methods design. Trials were randomized using a custom randomization tool in R Shiny (R Core Team), a system that allows researchers to generate complex, custom, and reproducible randomization sequences for clinical trials [14]. Clinical trials were randomized to recruitment strategy intervention arms of either CA, registry and patient engagement platforms (ResearchMatch [Vanderbilt University Medical Center], Stanford REDCap registry [Stanford University], UCSF CARE registry [University of California, San Francisco], and Studypages [Yuzu Labs PBC]), social media ads (Google [Google LLC] and Facebook [Meta Platforms, Inc], which later transitioned to BuildClinical [BuildClinical LLC] solely), or the control arm (usual recruitment). Recruitment began in January 2023 and was completed in April 2025. Eligible trials included adult, cardiometabolic clinical trials within the United States that were actively recruiting for at least the next 6 months, with additional time allowed as leeway. To participate, trials were expected to have the capacity to adopt any of the intervention strategies offered. Trials were identified through direct outreach by TOTAL’s PIs to professional colleagues and through announcements distributed via clinical listservs, including those of organizations such as PPD (Pharmaceutical Product Development) and similar groups. Sample size calculations showed adequate power if randomizing 36 sites with an enrollment rate of at least 2 patients per month.

The full study protocol is available on Contemporary Clinical Trials [15]. The statistical analysis plan is available at Open Science Framework [16].

#### TOTAL Project Intervention

The intervention period started in January 2023 and ended in February 2026. Participating trials received their allotted intervention for up to 6 months. Data were collected at baseline (0‐1 month), midintervention (3‐4 months), and end (6 months). Qualitative data were collected through semistructured interviews via Zoom (Zoom Video Communications, Inc) with trial PIs or clinical research staff, and quantitative data were collected through recruitment data. Additionally, observational data were collected throughout the intervention period through the TOTAL team’s supportive role in using the interventions. All of the trials randomized to a recruitment intervention had the support of a TOTAL staff member remotely to help them use the platforms or become connected to health organizations, while the participating trials managed direct contact with their research candidates.

The CA was a fully remote adaptation of Morehouse’s established CA role [17] and was employed by a TOTAL staff member who served as an intermediary between trial teams and community institutions. The CA used Airtable (Airtable, Inc), a cloud-based platform that enables teams to organize data, manage projects, and automate workflows [18]. Airtable was used to document and keep track of the surrounding, relevant community trials that have been contacted and followed up. The CA served as a liaison between the participating trials and community organizations, so that they could advertise the trials among their members. CAs would also help to edit trials’ recruitment materials to ensure that they were culturally inclusive and accessible to differing health literacy levels.

Registry and patient engagement platform usage was supported by a TOTAL staff member, and access depended on the type of trial, so each trial used anywhere from one to all of the platforms. The platforms were chosen based on their affordability and accessibility to general clinical trials. Stanford REDCap registry was available to Stanford trials and affiliates and included about 11,280 already-established, consented patients [19]. UCSF CARE registry provided access to connecting with Asian American, Native Hawaiian, and Pacific Islander populations in research, with more than 10,000 enrollees nationwide. This registry originated in the San Francisco Bay Area, and its current strength remained concentrated in this area during the time of the study [20]. ResearchMatch was also a national registry with over 156,000 consented patients and was free to use for partners, which included most academic institutions [21]. Studypages was a patient engagement platform and provided in-kind access to participating TOTAL trials during the intervention. This was an AI-driven, HIPAA (Health Insurance Portability and Accountability Act)-compliant platform designed to streamline clinical trial recruitment, participant management, and trial operations [22]. Among the 9 trials randomized to the registry and patient engagement platform arm, these platforms were used at these rates: Studypages (n=9), Stanford REDCap registry (n=4), ResearchMatch (n=8), and UCSF CARE registry (n=6).

The digital advertising intervention was implemented in 2 phases, consisting of an initial in-house phase managed by the TOTAL study team using Google Ads and Meta (Facebook) platforms and a subsequent vendor-managed phase conducted through a certified digital recruitment platform (OpenClinica, LLC, formerly BuildClinical). During the in-house phase, advertisement development followed a standardized approach in which a single IRB-approved core advertisement was created per study and adapted across multiple platform-specific formats. These adaptations included variations in layout, text length, and display format required by Google and Meta advertising specifications, such as headline variations, short and long descriptions, and image-based placements, rather than distinct creative concepts. For example, Google Ads campaigns required multiple headline and description variants within a single campaign structure, while Meta ads used a primary text, headline, and image combinations derived from the same core messaging. All advertisements contained consistent study messaging, including a brief description of the trial, eligibility framing, and a call to action directing users to learn more. Ads were deployed in the digital environment, such as search results and social media feeds, and functioned as passive recruitment tools. No direct outreach to potential participants was conducted through the advertising platforms. Instead, individuals self-initiated engagement by clicking on advertisements and navigating to study-specific landing pages.

Landing pages were hosted on secure Qualtrics (Qualtrics, LLC) or trial-specific platforms and served as the primary mechanism for participant engagement. These pages included study information and a structured interest form through which individuals could voluntarily submit contact information for follow-up by study personnel. Access to submitted information was restricted to authorized research staff. Campaign setup during the in-house phase required manual configuration within each platform, including selection of campaign objectives such as website traffic, geotargeting parameters aligned with trial catchment areas, and scheduling based on IRB approval timelines. To enhance relevance to target populations, advertisements incorporated imagery designed to reflect the demographic characteristics of populations served by participating trials. However, the ability to iteratively modify content was limited by IRB requirements, as all advertising materials required prior approval before deployment.

Following the transition to the vendor-managed phase, campaigns incorporated a broader range of advertisement creatives and optimization strategies. In contrast to the single-core ad approach used in the in-house phase, vendor-managed campaigns deployed multiple distinct creatives per trial, including approximately 11 to 22 static advertisements and 2 short video advertisements per campaign. Video creatives consisted of rotating slide-based formats derived from static ad components, typically 8 to 16 seconds in duration. Campaign budgets were standardized at approximately 1000 dollars per trial per month for media spend. Vendor-managed campaigns also enabled more advanced targeting and delivery optimization. Across these campaigns, advertisements generated over 641,000 impressions and 15,371 clicks, resulting in 1221 total leads, of which 685 (56%) were deemed eligible for follow-up. As in the in-house phase, participant contact was initiated only after individuals voluntarily submitted their information through study landing pages, in accordance with IRB-approved recruitment procedures.

### Data: Qualitative Interviews and Analysis

Interviewees were limited to participating trial representatives who had oversight or were heavily involved with their trials’ recruitment (ie, PIs, clinical trial managers, and clinical trial coordinators) and were chosen by their trial teams. Semistructured qualitative interviews focused on trial perspectives regarding recruitment strategies. The questionnaire guided discussions and included topics around diverse recruitment impacted by resources available, accessibility, staff capacity, staff expertise, and local populations. Refer to Multimedia Appendix 1 for the qualitative interview guide.

### Qualitative Data Analysis

We conducted rapid qualitative analysis using the Stanford Lightning Report framework [23], which organizes qualitative data into “plus” (what is working), “delta” (what needs to change), and “insight” (ideas or suggestions) domains. First, interview transcripts and detailed interviewer notes were reviewed in full by both coders. Data were entered into a structured spreadsheet matrix aligned with Lightning Report categories. Interview recordings were transcribed and analyzed using primarily a deductive thematic approach based on preidentified domains from the Lightning Reports. Coders also considered emergent, inductive themes. Both coders independently coded each transcript, then met to compare codes, discuss discrepancies, and refine the coding as needed. Codes were generated from recurring ideas and meaningful statements in the transcripts, then grouped into broader categories and themes that captured patterns across participants’ accounts.

The coders discussed interpretations and reconciled differences through consensus meetings. Analytic memos, “Lightning Reports,” were generated to summarize key themes, insights, and interpretations. All coding decisions and representative quotes were documented to support auditability and transparency. This systematic and collaborative consensus-based approach was designed to maximize the reliability, validity, and reproducibility of our qualitative findings.

### Ethical Considerations

Stanford School of Medicine’s IRB determined that this study’s only involvement of human participants in the research activities would be in one or more of the categories that are exempt from the regulations of 45 CFR 46.102(d) and 21 CFR 50.3(c). Morehouse School of Medicine’s IRB deferred to Stanford’s IRB decision. The TOTAL project was determined exempt from categorization of human participants medical research, according to Stanford Protocol number 66454. Morehouse School of Medicine’s IRB deferred to Stanford’s IRB decision. Informed consent was waived. To participate in qualitative interviews, trial participants were asked to review the IRB-approved Exempt Research Information Sheet outlining their participation in the study, indicate their agreement to participate to the TOTAL representative, and continue with the interview process. As compensation, one interviewee from each team was offered a US $20 gift card per qualitative interview completed, up to 3 interviews per trial.

## Results

### Participants

A total of 37 sites throughout the United States were randomized due to the capacity of resources for this study. A total of 35 were interviewed through a qualitative assessment due to the early withdrawal of sites. Trials were represented by PIs (n=1) and clinical trial managers and coordinators (n=35), as one of the PIs was present with another research staff member. A total of 50% (18/35) of participants did not respond to demographic questions. This was a diverse sample including perspectives from women (n=15), men (n=2), nonbinary (n=0) individuals, as well as Asian (n=2), Black (n=2; both identified as Hispanic in ethnicity), and White (n=13) participants. The time of interviews averaged between 9 and 11 minutes. Participants represented 35 of 37 participating trials, including 5 trials that withdrew from participation in the TOTAL project. Reasons for withdrawal included trials ending enrollment preemptively, sponsors closing out trials preemptively, lack of staff capacity, and lack of compensation for additional recruitment and data transfer duties.

### CA

Qualitative data regarding the CA intervention are presented in Table 1. The CA intervention primarily functioned to extend trials’ reach beyond their existing institutional networks. The CA established new connections with local churches, local businesses, neighborhood health clinics, nonaffiliated medical offices, and professional networks of cardiologists and other physicians, as well as race- and ethnicity-focused health organizations. Trial teams reported that, given their limited staff capacity for proactive community outreach, the CA filled a critical gap by initiating and maintaining these external relationships with minimal burden on staff workflows.

Recruitment outcomes associated with the CA were mixed. Some trials perceived little change in overall diversity or reported uncertainty about whether outreach efforts were yielding responses. One trial notes a meaningful increase in enrollment of Asian American participants, attributing this to referrals from a cardiologist who engaged through the CA, with a diverse patient panel and access to networks in the Asian American local community. However, similar gains were not observed for other racial and ethnic groups. Trials also highlighted ongoing structural constraints, including hospital location, which narrows eligible patient pools, availability of alternative treatment options, and limited budget for broader community advertising, which constrained the extent to which CA-generated ideas and partnerships could be fully implemented within the current trials.

**Table 1.** Thematic analysis of community ambassador.

Theme and sample quotes	Summary
Recruitment yield	
“The first two patients that [the community ambassador] sent had an exclusion and didn’t qualify. But [the community ambassador] said that they are really interested. It’s not a difficult-to-enroll study, so we look forward to recruiting more diverse patients from there. [The community ambassador] also reached out to a reverend from a church from a church association that is also in contact with a lot of health centers.” “I know [the community ambassador] has been reaching out to organizations in different mediums, but I’m not too sure if they’re getting any responses back.” “It has worked pretty well for us. We have been able to increase diverse recruitment, specifically with Asian participants, but have not seen much increase for other groups such as Hispanic and African American participants.” “Diversity through this intervention stayed the same.”	Participating trials reported mixed results in recruitment yields, with one trial noting higher recruitment rates from specific race and ethnicity groups compared to others. Other trials noted that diversity rates stayed the same.
User experience	
“It was quite easy to adapt [to the intervention]. [The community ambassador] helped us to identify more potential participants for our study.”	A participating trial described adapting to the community ambassador intervention as straightforward, with the ambassador providing valuable assistance in identifying potential participants.
Cost and resources	
“I wish we had a big budget for community advertising. Things like commercials, billboards, or direct-to-the-patient advertising rather than having to go through a clinic.” “Some of the ideas from the community ambassador, we can’t currently implement because that would require a new contract, study design, and additional IRB approval, but they are great ideas for our next studies when we do have the resources to add them.”	Budget and resource constraints limited the implementation of community advertising methods. The community ambassador shared innovative ideas, but one trial team was not able to implement them into their current trial due to additional effort and burden (ie, renewing contracts, study design changes, and IRB^a^ modifications).
Location	
“The hospital location may be difficult for people to come in for treatment.”	Hospital locations posed a barrier, making it difficult for potential participants to attend treatment visits.
Staff capacity	
“I haven’t had to make any changes to my work tasks to make use of this intervention.” “It didn’t take any extra effort on our part, with regard to using any specific kind of portal or referral service. So that was helpful. It didn’t feel like a burden.”	Staff capacity demands were minimal, with no need for workflow changes or additional effort to use the intervention, which participants found helpful and nonburdensome.
Study design	
“I would say the challenge for recruitment is that the patients themselves are quite difficult to find. Even to approach the study. Because there is a different treatment option available. So when patients are referred to us for this study [treatment], they had to have declined or not qualified for the other potential treatment option. So there are very few people who are receiving referrals to even discuss the study with.” “We’re really targeting people with [a specific heart disease], which tends to affect women and minorities more often than white and male. I don’t think the reason why we’re seeing that phenomenon is that we’re focusing our recruitment/enrollment on these types of clinics, I think it’s just a nature of the disease process that we’re studying.”	Study design created recruitment challenges through narrow patient pools. On the other hand, disease epidemiology can naturally impact enrollment of underrepresented groups.
Community connection	
“I have appreciated the [community ambassador’s] work. I haven’t received emails lately, but [they] worked a lot on having those ads translated and reached out to a lot of groups for fellows, residents, and cardiologists. One of them is based in Oakland, who is a cardiologist. Thanks to [the community ambassador], [the physician] has been really interested, looking to refer three to four patients, and has our inclusion/exclusion criteria. [The physician] is also having his assistants look out for patients. He thinks [this trial] will benefit his patients, and he has a lot of diverse patients.” “So maybe they haven’t found the right groups of interest. Maybe changing the focus from generic community settings like churches and clubs, to more neighborhood health clinics, non-affiliated medical offices, or free clinics.” “[In our institution], I wish we had a more established way to connect to cardiologists in the area to open up exposure to more potential patients.” “It would be helpful to pre-identify places that could be approached for outreach. Because I think [trial teams] are not really aware of the various community services and locations.”	Participating trials valued community ambassadors for targeted physician connections and referrals. Some noted varied communication rates, suboptimal venue targeting, and gaps in institutional networks and premapped partnerships.
Sustainability	
“It’s been great, the community ambassador has given us a lot of ideas that we will consider for our next studies.”	A participating trial viewed the community ambassador intervention as helpful for future studies, generating valuable ideas.

a IRB: institutional review board.

### Registry and Patient Engagement Platforms

Qualitative data regarding the registry and patient engagement platforms intervention are presented in Table 2. Trial teams generally described registry and patient engagement platforms as straightforward to implement and easy to learn, particularly when accompanied by recruitment-focused technical support. They appreciated having dedicated staff or support teams associated with these tools to assist with troubleshooting and outreach, and noted that institutional registries, such as those embedded in academic medical centers, facilitated contact with participants who already had an established, trusting relationship with the institution.

Recruitment yield from these platforms was variable. Some trials reported modest gains in enrollment and a slight increase in participation from underrepresented groups, while others observed limited impact and attributed the low yield to the characteristics of their local population or broader regional factors. Investigators emphasized the value of registries that explicitly focus on diverse populations and expressed interest in accessing a broader range of such platforms.

Several structural constraints limited the potential of registry-based strategies. Teams underscored that geographic distance to the trial location and the need for frequent in-person visits constrained participation, particularly for more diverse communities living farther from academic centers. Language inaccessibility was also identified as a critical barrier to recruiting and retaining non–English-speaking participants, with trials expressing a desire for more extensive language services but citing concerns about cost. Across settings, cost-free or institutionally supported tools, such as the no-charge components of the TOTAL initiative, were viewed as essential for uptake, given limited budgets and staff time dedicated to recruitment.

**Table 2.** Thematic analysis of registry and patient engagement platform.

Theme and sample quotes	Summary
Recruitment yield	
“Stanford REDCap registry yielded the most already-established patients to the trial.” “We definitely noticed an increase in diverse enrollment, though we hoped for a little more.” “Not seeing many results, but it could be because of the location population.”	Registries were helpful but variably productive. Using a registry with many already-established patients posed modest gains in rates of diverse recruitment. At the same time, trials noted that recruitment output was limited or inconsistent in certain settings and national regions.
User experience	
“Easy to implement.” “These tools were new to me, but I was able to learn easily, especially with additional guidance from the TOTAL team.” “There were no major barriers to getting the trial started.”	Participating trials reported a smooth, low-burden user experience with these tools, describing them as easy to implement and quick to learn, especially with external recruitment-specific support.
Cost and resources	
“TOTAL being free of charge was a huge plus, and we would not have opted in if the external support or registry platforms cost money.” “My advice to other researchers is to utilize the different registry tools, especially those offered by your institution.”	Participating trials valued cost-free tools and institutional resources as key enablers for recruitment, with TOTAL’s^a^ no-charge support being a major factor in their decision to participate. They recommended that other researchers prioritize free or institutionally provided registry platforms to maximize reach without straining budgets.
Language access	
“It would be helpful to have language services, but I’m not sure how much it would cost to have for recruitment and during trial participation.” “It would be helpful to have more language services and support in recruitment and in the study. It’s hard to recruit non-English speakers, ensure they’re making an informed decision, and retain them in the study if we don’t have the language capacity.”	Language inaccessibility was a critical gap in recruitment and retention, particularly for non-English speakers. Trials expressed interest in expanded language support but raised concerns about the associated costs.
Location	
“It is difficult to increase diversity when more diverse populations live further out from [the institution], especially when they would need to come to the hospital often.” “There are factors to keep in mind, such as the population in the Midwest, willingness to join a trial, and demographics of the city hospital versus satellite sites. This goes beyond the trial itself.”	Geographic and demographic barriers to diversity existed, noting that diverse populations may often live farther from trial sites, deterring frequent hospital visits. Trials also pointed to broader regional factors such as participant willingness and differences between central hospitals and satellite sites.
Staff capacity	
“Not much of a burden at all to implement registries.” “It was easy to implement and check on the platforms.”	Participating trials described implementing and monitoring registries as requiring minimal staff effort, viewing the process as straightforward and easily manageable within existing workflows.
Study design	
“This is a difficult study to recruit for in general. We ask a lot from participants. We require weekly visits several times per year for blood draws, with no payment.” “We would potentially use Studypages in the future, but recruitment strategies are typically decided by the PIs or sponsor.”	Study design features created inherent recruitment barriers. Trials noted that PIs^b^ or sponsors typically dictate the study design, so recruitment platform usage highly depends on their decision and awareness of strategies available.
Current events	
“TOTAL has an important initiative, but we did not notice an impact on diverse recruitment rates. Some of the patients were already consented and had queued to visit, but had been fearful of risking deportation in the area.”	Participating trials noted that while the TOTAL initiative held value, its impact on diverse recruitment was muted by external factors.
Diverse participant-focused platforms	
“It would be helpful to access more variety registries that have a specific focus on diverse populations, and are likely to yield diverse participants.”	Participating trials underscored a need for platforms explicitly designed to reach underrepresented groups. One of the only diverse participant-focused registry platforms accessible to most trials was UCSF^c^ CARE.

a TOTAL: Trial of Sites to Increase Diversity in Clinical Trials.

b PI: principal investigator.

c UCSF: University of California, San Francisco.

### Social Media Ads

Qualitative data regarding the social media ads intervention are presented in Table 3. Initial efforts to recruit underrepresented populations through social media ads (Google and Meta) generated a high volume of responses; however, many were either ineligible, unresponsive, or unrelated to the study, including spam entries and inquiries from individuals seeking employment. Trials also noted substantial operational challenges stemming from evolving platform policies on health-related advertising, which frequently led to ads being paused or rejected because of verification issues and stringent content requirements.

Transitioning from internally managed campaigns to partnering with BuildClinical introduced several important facilitators. BuildClinical’s expertise in health-related digital advertising helped teams navigate complex advertising policies and complete verification processes, substantially reducing interruptions caused by ad rejections. Trial teams reported that the intervention was relatively easy to integrate into existing workflows and that technical issues, such as problems with eligibility surveys, were resolved efficiently with support from both BuildClinical and the external recruitment team.

Despite these improvements, recruitment yield remained mixed. Social media campaigns expanded overall reach and increased exposure among diverse audiences, but the proportion of respondents who ultimately met eligibility criteria and enrolled in the trials was modest, reflecting substantial attrition from ad views to survey completion and eligibility. Trials also described institutional barriers to fully leveraging this strategy, including delays in executing contracts, processing purchase orders, and obtaining vendor approvals, which slowed startup and limited the continuity of advertising efforts.

**Table 3.** Thematic analysis of social media ads.

Theme and sample quotes	Summary
User experience	
“It was easy to integrate the intervention with the TOTAL team.” “Any issues with BuildClinical were resolved easily, like the eligibility survey, especially with additional support from the TOTAL team.”	Participating trials reported a positive user experience, describing the intervention as easy to integrate into existing workflows. They highlighted that any technical issues were quickly resolved, especially with external recruitment-specific support.
Eligibility criteria	
“If done 6 years ago, it would’ve been a well-recruiting study. But now patients are responding well to medical therapy instead of wanting to join invasive studies such as this.” “Slow rates of recruitment, especially because many did not want to join an invasive trial.” “Recruitment rates in general has its ups and downs, especially because our recruitment must come exclusively from [a specific occupation and department]. It would generally be best to reach out directly to them or through the funder.”	Strict participation requirements and narrow eligibility criteria posed major constraints on recruitment. Invasive procedures and alternative treatments available reduced the willingness to enroll.
Increased outreach but low enrollment yield	
“There’s been a surprising amount of views of the ad, but the rate at which people click into the survey is significantly lower, and the rate at which people are eligible for the study is even lower.” “With TOTAL’s ads, there has been somewhat of an improvement in reaching diverse candidates; however, there was not as much overall enrollment of diverse participants. Some of them were not eligible, and oftentimes, the surrounding population itself is not diverse.” “We are also getting so [many] spam responses on the ad Qualtrics form, but this was minimized after activating the human-authorization setting.”	Ads expanded reach, including to more diverse audiences, but generated few truly eligible participants. Engagement dropped off at each step (from views to clicks to eligibility), and spam responses further reduced yield, even though technical filters could partially mitigate this issue.
Ad policies and content	
“We have not been able to do the ads because of Google Ads’ policy limitations and constant changes.” “This highlights a barrier when using ads when it’s specific to stem cell research. You have to be careful with wording on the ad page and Qualtrics screening survey to really follow the ad regulations.” “On the other hand, some patients actively look for stem cell research to participate in, but sometimes the ads have to be too generalized, so patients can’t always tell what they are looking at.”	Advertising policies were described as a significant barrier, noting that platform rules and frequent changes prevent or stall ads from running. There was an emphasized need for highly cautious, precise wording in advertisements and screening surveys to comply with regulations, while being detailed enough to get their point across.
Motivation	
“We liked the idea of TOTAL, having a team to support diverse recruitment, and wanted to participate.” “Staff are interested in the intervention and want to increase diversity in the study.”	Participating trials emphasized a strong, proactive commitment to improving diversity in clinical trials, valuing external support specifically aimed at recruiting underrepresented groups.
Prioritization of other strategies	
“There were zero hits so far on BuildClinical. BuildClinical hasn’t been prioritized as much because we are already using another dashboard that has been more effective.”	One trial perceived BuildClinical as a lower priority than their other recruitment strategies, because it produced no recruitment yield and competed with an existing tool, which they viewed as more effective.
Satisfaction with strategy	
“It’s been going pretty well.” “It has been alright, not too much impact on recruitment.”	Participating trials expressed moderate satisfaction with the strategy, describing it as functioning smoothly but yielding limited, measurable impact on recruitment.

### Usual Recruitment

Qualitative data regarding the usual recruitment arm are presented in Table 4. Most trials relied on physician referrals as their primary recruitment strategy, with enrollment shaped by existing electronic health records, referral networks, institutional reputation, and the finite pool of eligible patients within their hospitals or affiliated practices. The recruitment teams noted that they often exhausted their usual patient pools, particularly when eligibility criteria were narrow or when there were simply fewer eligible patients available at the institution. Staff turnover occasionally disrupted recruitment continuity by necessitating retraining, although teams generally reported being able to maintain recruitment efforts with adequate institutional support.

Several trials described distinctive approaches layered on top of usual recruitment. One trial joined a gamified “recruitment Olympics” competition across multiple hospitals, which substantially increased motivation among clinicians and led to a surge in referrals from nurse practitioners and physicians. Another trial achieved a notable spike in inquiries and enrollments by securing coverage through local news outlets and collaborating with a local sports celebrity; however, this type of strategy is highly contingent on the PI’s existing media and community connections. Despite these efforts, many trials reported that participant diversity remained largely unchanged, with enrollment continuing to be skewed toward White, non-Hispanic patients.

Timely communication with potential participants emerged as an important operational factor, as delays of more than a week often resulted in loss of interest or contact. In addition, some usual recruitment approaches imposed unintended access barriers, which further narrowed the eligible pool. Trials also highlighted the limitations of phone-based interpreter services, noting that while they facilitate basic communication, they do not fully capture the emotional and cultural nuances of in-person interactions that are critical for building trust with non–English-speaking participants.

**Table 4.** Thematic analysis of usual recruitment.

Theme and sample quotes	Summary
Recruitment yield	
“[Diversity of patients] has stayed the same. The majority are White, Non-Hispanic.”	Despite recruitment efforts, participant diversity remains stagnant, with enrollment continuing to skew toward White, non-Hispanic populations.
Eligibility criteria	
“A barrier to participation is that our study requires participants to have an iPhone.”	Restrictive eligibility criteria, such as device requirements, can inadvertently narrow the participant pool and introduce barriers to broader, more diverse enrollment.
Language access	
“Having interpreters onsite, in person, would help. A lot of the time, we have interpreters over the phone. It is doable. But it can be difficult to make sure you’re being heard correctly. For a lot of cultures, it’s not just verbal communication. There can be a lot of emotions involved, and that can get cut off when you’re over the phone.”	Remote interpretation, while functional, can fall short of meeting the full communicative and cultural needs of non–English-speaking participants, highlighting the value of in-person language support for more equitable, accurate, and humanizing engagement.
Communication	
“When we are not able to reach out to patients within a week, they often fall off.”	Timely outreach is critical to recruitment yield, as delays of even a week risk losing prospective participants before enrollment can be completed.
Gamified recruitment incentives	
“We had a recruitment summer Olympics, competing with five other hospitals, and you get more points per patient consented and randomized, or if you answer a query. This made it fun and motivating. We got 52 referrals from NPs and physicians.”	Gamified competition across sites can meaningfully boost recruitment motivation and referral volume by transforming routine enrollment tasks into an engaging, goal-oriented experience.
News outlets and local figures	
“We had a press release. That was a huge success. 20 to 25 patients called the practice to refer themselves or had to ask their cardiologist to connect to us. We had a news outlet. [Our PI] interviewed with one of the local news channels, and it was posted to Facebook and LinkedIn. Within the first week, we had 15 participants, and they continued to trickle in. Not as many as at the beginning, but it was a lot at the start.” “With the presence of cardiac arrest [in sports], [a local celebrity in professional sports] and our PI did a local training with the community. I think our PI has [motivation] to talk about this condition [with the community], so it really stems from our PI reaching out to the marketing team.”	Strategic use of media (ie, press releases, local news features, and high-profile community events) can generate significant recruitment surges, particularly when driven by a motivated PI^a^ who actively cultivates relationships with marketing, public, and community platforms.
Physician referrals, reputation, and patient pools	
“Generally, our recruitment is through [physician] referrals, so it depends on our PI to reach patients and other doctors.” “We tend to exhaust our patient pool through usual recruitment.” “When there are fewer eligible patients in the hospital, that is out of our control.” “[Rate of enrollment] often has to do with how well-established our cardiology practice is.”	Physician referrals are the most used recruitment strategy. Recruitment success and challenges are shaped by interconnected structural factors, such as physician referral networks, institutional reputation, and finite patient pools.
Staff turnover and training	
“There has been turnover in our team, so learning the recruitment process can take time, but we’ve been doing a pretty good job with keeping up.”	Staff turnover disrupts recruitment continuity by requiring repeated retraining, though teams can maintain momentum through institutional support and commitment.

a PI: principal investigator.

### Barriers Across Interventions

Across all recruitment approaches, trials identified a set of persistent barriers that limited their ability to enroll diverse participants. Transportation challenges, lack of financial or other incentives, and study designs that required invasive procedures or frequent, time-consuming visits reduced the willingness and feasibility of participation for many patients. Restrictive eligibility criteria, such as requirements for specific devices (eg, iPhones), narrowly defined clinical conditions, or particular prior medical histories, further constrained the pool of eligible participants and disproportionately excluded some groups.

Regulatory and administrative processes also posed significant obstacles. The cost of revising recruitment materials to better represent diverse populations, combined with lengthy review timelines, discouraged frequent updates. Single institutional review board (sIRB) review was described as particularly slow and resource-intensive: sponsors commonly required sIRB approval before local institutional review, with sIRB review often taking one to 2 months, followed by an additional month for local IRB acknowledgment or secondary approval. To manage fees and reduce the number of submissions, many trials bundled multiple pending protocol and materials changes into single sIRB submissions, which unintentionally prolonged the overall modification cycle and delayed the implementation of diversity-enhancing recruitment strategies.

### Role of Systems and the Institution

Trials emphasized that institutional infrastructure plays a critical role in supporting diverse recruitment. They envisioned dedicated, HIPAA-compliant recruitment teams embedded within their institutions that could cultivate and maintain community partnerships, conduct outreach on behalf of study teams, and proactively engage potential participants identified through registries or electronic health records.

Several teams suggested leveraging patient-facing portals (eg, MyChart; Epic Systems Corporation) to allow individuals to opt in to receive notifications about relevant research opportunities based on their medical history and prior visits. Trials also proposed developing registries of in-network health care professionals who could be mobilized to discuss trials with their own patients. Across interviews, investigators noted that patients appeared more motivated to participate when recruitment occurred through a trusted physician, underscoring the importance of integrating recruitment efforts with existing clinical relationships.

## Discussion

### Principal Findings

Findings are based on qualitative interviews across multiple interventions and participating trials. All participating trials emphasized the importance of supporting increased diversity in clinical trials and underscored the value of external, recruitment-focused support. The TOTAL team was viewed as particularly helpful in taking on tasks that trials often struggle to sustain, such as digital advertising, registry use, and community outreach, given chronic constraints in time, staffing, and recruitment-specific expertise. At the same time, the TOTAL team’s inability to serve on local IRBs limited their role in participant screening and direct access to medical records, reinforcing the need for institutionally embedded, HIPAA-covered recruitment infrastructure.

Across interventions, investigators highlighted interest in moving beyond exclusive reliance on physician referrals toward a more diversified recruitment portfolio that includes electronic health records, institutional and external registries, social media campaigns, and community-based outreach. However, the effectiveness of these strategies varied. Registry and patient engagement platforms and social media tools were generally easy to implement and integrate into workflows, and similar to a systematic review of registries, this study found that the actual benefits of registries depend heavily on their design, quality assurance, and the extent to which they are embedded into trial processes [24]. CA efforts showed promise in building new relationships with physicians, churches, and community organizations, yet improvements in diversity were uneven and sometimes confined to specific racial or ethnic groups, such as Asian American participants in one trial. This is similar to other findings in a study of community-responsive health systems, which documented that gains in inclusion and representation often occur unevenly across racial and ethnic groups, reinforcing the risk that engagement efforts benefit some subpopulations more than others [25].

The findings point to structural and design-related barriers that cut across strategies. Geographic distance from academic centers, lack of transportation and incentives, invasive or time-intensive protocols, narrow eligibility criteria, and language inaccessibility all constrained who could realistically participate. Trials repeatedly emphasized that patients were more likely to enroll when approached by trusted clinicians and when logistical burdens, such as device requirements, visit frequency, and language services, were proactively addressed. These observations support a model in which institutions develop centralized, HIPAA-compliant recruitment teams that can partner with study staff, leverage electronic tools, maintain community and clinician networks, and help tailor recruitment approaches to local populations. The Trials Learning Health System model aligns closely with these ideas, emphasizing improved recruitment by continuously tracking enrollment data and using that information to guide targeted interventions while trials are ongoing. It also emphasizes that trial data should include recruitment methods, timing, and success tracking so the system can learn what works across sites and populations [26].

Fostering diversity in clinical trials requires a need for evidence and practical approaches, and trial teams should work with community members and stakeholders to remove barriers before enrollment [27]. Trials can be better equipped by budgeting in advance for diversity-enhancing strategies and IRB-related costs, selecting combinations of recruitment methods that align with the study design and target populations, and building flexibility into implementation to adjust in response to real-time feedback. Practical steps include using technologies compatible with a range of devices, supplying or loaning needed equipment, providing transportation and incentives, accommodating participant schedules, and investing in language and cultural mediation. Many of these adaptations depend on sponsor and institutional commitment to funding recruitment as a core scientific activity rather than an ancillary task. Finally, while this study focused on enrollment, future work should examine how similar accessibility and trust-building factors influence retention of diverse participants over the course of a trial.

At a broader level, our findings align with critiques of academic medical centers as both central actors in the clinical trials enterprise and contributors to persistent inequities in trial participation. Fragmented or short-lived diversity, equity, and inclusion (DEI) initiatives, often tied to grant cycles or publication goals rather than sustained community engagement, are poorly suited to addressing the structural barriers that underrepresented populations face in accessing trials [28]. Limited investment in roles such as community liaisons, patient navigators, and CAs can leave institutions without the relational infrastructure needed to translate stated commitments to diversity into practice [29]. Given that academic recruitment often draws from affiliated hospitals and clinics that may be geographically and socioeconomically distant from diverse communities, intentional outreach and restructured funding mechanisms are needed.

Meaningful change will thus require institutional equity action plans that explicitly address community partnerships, sustained funding for recruitment and engagement roles, and protection of DEI-focused structures in the face of political and fiscal pressures [30]. In parallel, the concept of a Trials Learning Health System (LHS) offers a framework for continuous improvement in recruitment and inclusion [31]. Similar to LHS models in health care delivery, a Trials LHS would emphasize ongoing data collection on enrollment metrics, routine sharing of lessons learned, and rapid-cycle testing of improvement interventions [26]. Use of the Lightning Report approach demonstrates how embedded, rapid qualitative assessment can help identify context-specific barriers and facilitators in near real time, allowing trial teams to refine strategies midimplementation rather than only in retrospect [32].

### Limitations

This study has a primary limitation related to the sample. Participating trials were predominantly located at academic medical centers, with few independent or community-based trials. This institutional skew may limit the transferability of findings to settings where infrastructure, patient populations, and resource constraints differ substantially from those of large academic institutions. Common reasons for trials withdrawing from the TOTAL project include: paused trials, sponsor decision to end the enrollment period early, and meeting recruitment numbers early. In addition, the TOTAL team could not provide face-to-face support as the project was not designed as human subjectsparticipants research or a HIPAA-covered activity. The TOTAL team also sought connections with more diverse population-focused registries, but they were often not ready for launch within the timeline of the project, constraining opportunities to engage underrepresented groups through established community-trusted platforms.

Additionally, this study was limited by its reliance on predominantly academic medical center-based trials, which may reduce generalizability, and by structural barriers such as limited resources, restrictive eligibility criteria, language access gaps, and local population constraints that affected recruitment and implementation. In the future, more robust recruitment teams can build on the findings of the TOTAL project to inform more scalable and effective approaches to diverse trial enrollment.

### Conclusion

Diversity in clinical trials is essential for ensuring that evidence on safety and effectiveness is applicable across populations and for reducing inequities in treatment outcomes. The qualitative findings from this work highlight that while trials are highly motivated to improve representation and are willing to adopt multiple recruitment strategies, structural barriers, institutional constraints, and variable yields across approaches continue to limit progress. By investing in centralized, HIPAA-compliant recruitment support, strengthening community and clinician partnerships, and using rapid, data-informed methods such as Lightning Reports to iteratively refine recruitment strategies, institutions and sponsors can foster more inclusive trial environments that facilitate participation from underrepresented groups. In turn, more representative enrollment will support a more robust understanding of treatment effects across diverse populations and help build a stronger foundation for equitable clinical care in the future [33-35].

## Supplementary material

10.2196/86541 Multimedia Appendix 1 Qualitative interview guide.

10.2196/86541 Checklist 1 CONSORT checklist.

## Abbreviations

CA: community ambassador
CONSORT: Consolidated Standards of Reporting Trials
DEI: diversity, equity, and inclusion
HIPAA: Health Insurance Portability and Accountability Act
IRB: institutional review board
LHS: Learning Health System
PI: principal investigator
PPD: Pharmaceutical Product Development
sIRB: Single institutional review board
TOTAL: Trial of Sites to Increase Diversity in Clinical Trials
