# Supplementary material for: Diverse Recruitment for Clinical Trials Using Three Diversity-Enhancing Interventions: Qualitative Study
Source: JMIR Form Res. 2026 Aug 7;10:e86541. doi: 10.2196/86541 (PMC13451007; doi:10.2196/86541)
Supplement: Supplementary file 2 [file formative-v10-e86541-s001.docx]

## TOTAL Project: Interview Guide

**Interviewees**: Site Coordinators/Managers & PIs

**Timeline**: Baseline (0-1mo), Mid-Intervention (3-4mo), & End (6mo)

**Research Question(s):**

1. How acceptable is this strategy?
2. What is the perceived adaptability and complexity of this strategy?
3. What are the barriers and facilitators?
4. What is the cost of this strategy?
5. What factors influence decision-making, RE: continuing after the trial ends?
   a. Constructs:
      i. Baseline recruitment behavior
      ii. Awareness (of diversity in their trials)
      iii. Acceptability
      iv. Adaptation & Complexity
      v. Barriers and Facilitators
      vi. Adoption & Implementation
      vii. Cost
      viii. Maintenance factors

**Aim**: to understand the facilitators and barriers to improving diverse recruitment in clinical trials, and to see how each strategy may support diverse recruitment.

**Consent**:

- Exempt Research Information Sheet prior to the interview meeting
- Ask for verbal consent prior to recording and the start of the interview

### Questions:

***Baseline***

1. Background
   a. **Which populations do you think of when you think of diverse populations?**
   b. **Do those population groups typically live or work around your site’s location?**
2. Current State
   a. **How do you currently recruit participants?**
   b. **Do any of those specifically focus on recruiting diverse participants?**
3. Tracking/Awareness
   a. **How, if at all, do you track diversity of enrollment in your trials?**
4. Baseline
   a. **What, if anything, is your site doing to increase enrollment of diverse patients?**
      i. Prompts around diverse groups
         1. low-income, underserved
         2. race and/or ethnicity
         3. Disability
         4. Age
         5. Gender and/or sex identity
         6. Sexual orientation
5. Feasibility/Acceptance
   a. **How feasible would it be to adopt a diversity-enhancing recruitment strategy? (cost, staff expertise, staff capacity?**
   b. **Would diversity be a good thing to pursue even if it required changes to your recruitment infrastructure (cost) or more time committed to recruitment?**
   c. **What support would you ideally want at your institution to increase diversity in recruitment?**

***Mid-Intervention & End (+ additional questions below)***:

- Acceptability
  - **How acceptable has the intervention been?**
- Effectiveness
  - **What, if any, changes in enrollment have you seen as a result?**
- Barriers and Facilitators:
  - **What is helping you enroll more diverse patients? What is getting in the way of increasing diverse patient enrollment?**
- Adaptation
  - **What adjustments have you made in order to use the intervention? What would you need to change for it to work better?**
- Complexity
  - **How complex do you feel like this is?**
- Start-up
  - **How much effort have you needed to get this started**
- Cost
  - **How much effort have you needed to keep this going? What other resources have you used to make this work?**
- Maintenance
  - **What would be needed to continue these long–term results? How would you know you’re being successful in recruiting diverse participants?**
  - **How likely is this intervention to produce lasting changes in your recruitment of diverse patients? What about in 6 months from now - would you want to be using this intervention**

***Mid-intervention additional questions:***

- **How has enrollment been going?**
- **What have been your main strategies for recruitment? Have any of them noticeably increased recruitment?**
- **I see you’ve increased/decreased enrollment of diverse groups. What happened? How did you achieve this?**
- REGISTRY-SPECIFIC
  - **Which registry do you feel has yielded the most participants?**
- COMMUNITY AMBASSADOR-SPECIFIC
  - **Which community organizations do you feel have yielded the most participants?**
- SOCIAL MEDIA AD-SPECIFIC
  - **Which ads do you feel have yielded the most participants?**

***End of intervention additional questions***:

- Retrospective ease of uptake/Cost
  - **How easy/difficult was it for your trial to adopt this intervention?**
- Impact
  - **Did you notice an increase/decrease/same rates in diversity?**
- Site variation as a factor
  - **What do you think impacted recruitment success or challenges in recruitment, specific to the type of trial you are? [brand new site, well-established site, well-funded site, type of condition, etc]**
  - **What other factors impacted your success or challenges in recruitment? [current events, site infrastructure changes]**
- Dissemination/Implications/Future
  - **What do you think could have helped the recruitment intervention be more successful?**
  - **Now that you’ve done this, what advice would you give to other researchers starting to engage with this strategy?**
  - **What types of resources do you wish your institution had to enhance diverse recruitment?**
  - **Are there any other points about diversity & recruitment that you’d like to add?**
  - **Would they still want the service if they had to pay for it (Anekwe)? Value proposition.**
- USUAL RECRUITMENT-SPECIFIC
  - **What were your trial’s usual recruitment strategies? Did they change?**
  - **How did you do with diverse recruitment?**

***Demographic Questions for CRC/Manager/PI***

| **1) Role**  1 = PI  2 = Manager or Coordinator  3= Other team member (specify) | **2) How long have you been in this position (yrs)** | **3) Race**  1 = White  2 = Black or African American  3 = American Indian or Alaska Native  4 = Asian  5 = Native Hawaiian or Other Pacific Islander  6 = Other (specify)  7 = Multiracial  8 = N/A or prefer not to disclose | **4) Ethnicity**  1 = Hispanic  2 = Non-Hispanic  3 = N/A or prefer not to disclose | **5) Sex**  1= Female  2= Male  3 = Nonbinary  4 = Other (specify)  5 = N/A or prefer not to disclose |
| --- | --- | --- | --- | --- |
